# A Novel Epiphytic *Clonostachys chloroleuca* Strain that Effectively Controls Cucumber Anthracnose

**DOI:** 10.1264/jsme2.ME26058

**Published:** 2026-09-29

**Authors:** Alchemi Putri Juliantika Kusdiana, Yuta Hayakawa, Stephany Angelia Tumewu, Haruhisa Suga, Masafumi Shimizu

**Affiliations:** 1 The United Graduate School of Agricultural Science, Gifu University, 1–1 Yanagido, Gifu-shi, Gifu 501–1193, Japan; 2 Faculty of Applied Biological Science, Gifu University, 1–1 Yanagido, Gifu-shi, Gifu 501–1193, Japan; 3 Indonesian Rubber Research Institute, Banyuasin, South Sumatra 30953, Indonesia; 4 Institute for Glyco-core Research (iGCORE), Gifu University, 1–1 Yanagido, Gifu-shi, Gifu 501–1193, Japan

**Keywords:** biological control, *Clonostachys chloroleuca*, *Colletotrichum orbiculare*, cucumber, phyllosphere-associated fungi

## Abstract

Cucumber anthracnose is a destructive disease caused by *Colletotrichum orbiculare* that affects global cucumber production. Although plant-associated fungi are promising sources of biological control agents, their efficacy against cucumber anthracnose has not been established. In the present study, 108 spore-forming endophytic and epiphytic fungal strains were isolated from the leaves of seven plant species and screened for biocontrol activity against *C. orbiculare* using detached cucumber cotyledon assays and pot experiments. Three highly suppressive strains (GUSF-44, GUSF-66, and GUSF-153) identified in detached cotyledon assays were further evaluated in pot experiments, in which GUSF-66 exhibited the most consistent and pronounced disease suppression, reducing the lesion area by approximately 75%. A multilocus phylogenetic anal­ysis based on the internal transcribed spacer region, RNA polymerase II second largest subunit gene, and translation elongation factor 1-alpha gene sequences identified GUSF-66 as *Clonostachys chloroleuca*. Microscopic observations showed that this strain successfully colonized the cucumber cotyledon epiphytically. *In vitro* assays revealed that GUSF-66 produced lytic enzymes, including chitinase, β-1,3-glucanase, and protease, and inhibited the conidial germination and hyphal growth of *C. orbiculare*. Although some *C. chloroleuca* strains have been implicated in soybean root rot, GUSF-66 caused no visible symptoms in soybean plants. To the best of our knowledge, this is the first study to demonstrate the biocontrol potential of *C. chloroleuca* against cucumber anthracnose.

Plant diseases markedly constrain global food production, causing annual yield losses exceeding 30% and resulting in severe economic effects (Gai and Wang, 2024). Cucumber (*Cucumis sativus* L.) ranks among the most widely cultivated fruit-bearing vegetables (Che and Zhang, 2019). However, its productivity and quality are highly vulnerable to several phytopathogens. Anthracnose caused by *Colletotrichum orbiculare* (Berk. et Mont.) Arx (syn.: *C. lagenarium*) is among the destructive diseases affecting cucumber (Matsuo *et al.*, 2022). The pathogen infects leaves, stems, peduncles, and fruits (Roberts *et al.*, 2023), causing large losses in warm, humid conditions (Matsuo *et al.*, 2022). Under favorable environmental conditions, anthracnose epidemics may reduce cucumber yields by up to 60% (Conforto *et al.*, 2024), underscoring the economic importance of the disease. The management of cucumber anthracnose is largely based on the application of chemical fungicides, such as chlorothalonil, captan, thiophanate-methyl, iminoctadine tris (albesilate), and mancozeb. However, concerns regarding the unintended effects of excessive fungicide use on human health, non-target organisms, and the environment, as well as the development of fungicide resistance, have been raised (Islam *et al.*, 2024). The appearance of *Colletotrichum* species resistant to various chemical fungicides is well documented (Cortaga *et al.*, 2023). These concerns highlight the need for safer, environmentally sustainable, and effective alternatives to synthetic fungicides.

Biological control using beneficial microorganisms has emerged as a promising strategy to reduce dependence on synthetic fungicides and support more sustainable crop protection systems (Ayaz *et al.*, 2023). Previous studies exami­ned the use of microbial biological control agents (BCAs) against *C. orbiculare*. Most of these studies focused on bacterial and actinomycete BCAs isolated from soil and‍ ‍plant-associated environments, such as *Bacillus* (Nahar‍ ‍*et al.*, 2025), *Paenibacillus* (Haqyar *et al.*, 2026), *Pseudomonas* (Kim *et al.*, 2023), and *Streptomyces* spp. (Shimizu *et al.*, 2009; Ueno *et al.*, 2012; Chai *et al.*, 2022; Han *et al.*, 2026). Less attention has been devoted to fungal BCAs against *C. orbiculare*; however, several antagonistic fungi, including *Trichoderma* spp., hypovirulent binucleate *Rhizoctonia* spp., and plant growth-promoting fungi such as *Phoma* and *Penicillium* spp., have been reported to suppress cucumber anthracnose (Koike *et al.*, 2001; da Silva *et al.*, 2011; Hossain *et al.*, 2014; Elsharkawy *et al.*, 2015; Muslim *et al.*, 2019; Vani *et al.*, 2026).

Given their natural adaptation to plant-associated environments in which pathogen infection and colonization occur, plant-associated fungi inhabiting the phyllosphere or internal plant tissues are promising sources of novel BCAs (Bruisson *et al.*, 2019; Rakhalaru *et al.*, 2025). Endophytic and epiphytic fungi both establish beneficial interactions with plants and protect against plant pathogens through multiple mechanisms, including competition with pathogens, the production of antifungal metabolites, mycoparasitism, and the induction of host resistance (Fontana *et al.*, 2021; Oubohssaine *et al.*, 2026). Therefore, the examination of plant-associated fungi is warranted to facilitate the discovery of novel BCAs effective against foliar diseases, such as cucumber anthracnose.

This study attempted to isolate and characterize plant-associated fungi with biocontrol activity against cucumber anthracnose and identify promising fungal BCAs capable of suppressing the disease.

## Materials and Methods

### Isolation of epiphytic and endophytic fungi

The fungal isolates used in this study were obtained from an existing collection in our laboratory that had originally been established from rubber (*Hevea brasiliensis*) and six related Euphorbiaceae species to obtain a diverse collection of phyllosphere fungi. The healthy leaves of rubber (*H. brasiliensis*), Japanese mallotus (*Mallotus* sp.), African milk tree (*Euphorbia trigona*), croton (*Codiaeum variegatum*), cassava (*Manihot esculenta*), castor bean (*Ricinus communis*), and poinsettia (*Euphorbia pulcherrima*), collected in Gifu Prefecture, Japan, were used as sources for fungal isolation. To isolate epiphytic fungi, leaf segments were immersed in sterile distilled water (SDW) and sonicated for 30 s to dislodge surface-associated microorganisms. The resulting suspension was spread onto potato sucrose agar (PSA) plates. To isolate endophytic fungi, leaf segments were surface-sterilized with 2% (v/v) sodium hypochlorite for 1 min, followed by 70% (v/v) ethanol for 1 min, and then rinsed three times with SDW. Sterilized segments were homogenized with a sterile mortar and pestle, diluted in SDW, and plated onto PSA supplemented with chloramphenicol (50 μg mL^–1^). All plates were incubated at 25°C for 7 days. The emerging fungal colonies were purified by transferring hyphal tips to fresh PSA plates, followed by cultivation. This process was repeated until cultures were visually uniform. The agar plugs of purified fungi were stored in sterile 20% (v/v) glycerol at –80°C until used. Fungal isolates that did not form spores on PSA plates were deemed unsuitable as BCAs and discarded at this stage.

### Preparation of fungal inocula

*C. orbiculare* strain 104-T (MAFF240422) was used as the challenge pathogen. The strain was grown on PSA at 25°C for 7 days. Conidia were harvested by gently scraping the colony surface, filtered through three layers of sterilized Kimwipes to remove mycelial fragments, and suspended in SDW. The concentration of the conidial suspension was adjusted as necessary using a hemocytometer.

The epiphytic and endophytic fungal strains isolated in this study were grown on PSA at 25°C for 7 days. The conidia were harvested by gently scraping the colony surface, filtered through three layers of sterilized Kimwipes, and suspended in 0.01% (v/v) Silwet L-77 (Nippon Unicar). The concentration of the conidial suspension was adjusted as necessary using a hemocytometer.

### Detached cucumber cotyledon assay

To select potential BCAs against cucumber anthracnose, the disease-suppressive activity of a collection of fungal strains was assessed using the detached cucumber cotyledon assay described by Shimizu *et al.* (2009), with slight modifications. Cucumber seeds (*C. sativus* “Tokiwa Jibai”) were rinsed in 0.1% (v/v) Tween 20 for a few seconds, surface-sterilized with 1% (w/v) sodium hypochlorite for 3 min, and rinsed three times with SDW. The seeds were germinated on moist filter paper at 25°C for 2 days and then transplanted into plastic pots (5×5×5 cm^3^) containing a double-autoclaved commercial soil mixture (Saika-Ichiban; Ibigawa Kogyo) and covered with autoclaved vermiculite. The seedlings were grown in a controlled-environment chamber (25°C, 12 h light/12 h dark). Five days after transplanting, the cotyledons were detached from the seedlings and dipped in the conidial suspension (approximately 1×10^6^ conidia mL^–1^) of each fungal strain for a few seconds. As the control treatment, the cotyledons were similarly dipped in sterile 0.01% (v/v) Silwet L-77 solution. They were placed abaxial side down on 1.5% (w/v) water agar in 9-cm Petri dishes (four cotyledons per plate) and incubated in a controlled-environment chamber at 25°C for 1 day under a 12-h/12-h light/dark cycle. Two 5-μL droplets of the pathogen conidial suspension (approximately 1×10^6^ conidia mL^–1^) were then spotted onto each cotyledon, and the cotyledons were incubated for another 7 days. In this experiment, there were three replicate plates for each treatment (12 cotyledons per treatment).

The cotyledons were imaged using a digital scanner, and the lesion area was quantified using ImageJ software version 1.54 g (US National Institutes of Health, Bethesda, MD, USA; http://imagej.org). The percent reduction in the lesion area relative to the control was used to express disease suppression in the fungal treatments and was calculated as follows:% lesion area reduction=([*L_1_*–*L_2_*]/*L_1_*)×100, where *L_1_* and *L_2_* represent the mean lesion area in the control and treated samples, respectively.

### Pot experiments

Fungal strains that reduced the lesion area by more than 60% in the cotyledon assay were selected as primary candidates and subjected to pot experiments to confirm their efficacy on the true leaves of cucumber plants. After surface sterilization and germination as previously described, cucumber seeds were transplanted into 9-cm plastic pots that contained a double-autoclaved commercial soil mixture. The seeds were then covered with a small amount of autoclaved vermiculite. The plants were grown in a controlled environment chamber at 25°C under a 12-h/12-h light/dark cycle for 3 weeks. Approximately 1 mL of the conidial suspension (approximately 1×10^7^ conidia mL^–1^) of each primary candidate strain was then uniformly sprayed onto the fully expanded second true leaf of each cucumber plant using a glass atomizer. Control plants received only 0.01% (v/v) Silwet L-77. The plants were then incubated under the same conditions for 1 day and subsequently challenged with the pathogen, which was inoculated by applying twenty droplets (10 μL each) of the conidial suspension (approximately 1×10^6^ conidia mL^–1^) onto each treated leaf. After spotting the conidial suspension, each droplet was covered with a lens-cleaning tissue disc with a diameter of 5 mm. The inoculated plants were kept at 100% relative humidity and 25°C for 30 h and then placed in a controlled environment chamber (25°C, 12-h/12-h light/dark cycle) for 6 days. There were five replicate pots for each treatment, and the experiment was repeated three times. A disease assessment was performed by counting the number of lesions per leaf and measuring the total lesion area per leaf using ImageJ software version 1.54 g.

### Morphological characterization of GUSF-66

GUSF-66, which was selected in the pot experiment, was cultured on PSA at 25°C for 30 days in the dark, and the color and texture of its colonies were observed. The shapes of the conidiophores, phialides, and conidia were exami­ned using a BZ-X810 All-in-One Fluorescence microscope (Keyence).

### *In vitro* antagonism assay

The antagonistic activity of GUSF-66 against *C. orbiculare* 104-T was assessed using a dual culture assay. Mycelial plugs (5 mm in diameter) were excised from the actively growing margins of 7-day-old GUSF-66 and the pathogen colonies on PSA. One plug from each strain was placed on a PSA plate 6 cm apart and incubated at 25°C for 10 days. Plates inoculated with the pathogen alone served as controls. Antagonistic activity was quantified as the percent inhibition of radial growth as follows:% inhibition=([*R_1_*–*R_2_*]/*R_1_*)×100, where *R_1_* and *R_2_* represent the pathogen’s radial growth under the control and GUSF-66 conditions, respectively. Each treatment was conducted in three independent experiments, with three technical replicates per experiment.

### Light microscopy of the *in vitro* interaction between *C. orbiculare* and GUSF-66

The *in vitro* interaction between *C. orbiculare* and GUSF-66 was exami­ned by light microscopy as described by Shimizu *et al.* (2022) with minor modifications. Equal volumes of the *C. orbiculare* and GUSF-66 conidial suspensions, each adjusted to 5×10^4^ conidia mL^–1^, were thoroughly mixed. A 50-μL aliquot of the mixture was then placed on a thin layer of 2% (w/v) water agar that had been solidified on a slide glass, followed by an incubation in the dark at 27°C. *C. orbiculare* cultured alone under the same conditions served as the control. Five replicate slides were prepared for each treatment. One day post-inoculation (dpi), the samples were observed under a light microscope (Axio Imager A1; Carl Zeiss), and the germination rate of *C. orbiculare* conidia was assessed.

### Enzymatic activity assays

The ability of GUSF-66 to produce hydrolytic enzymes, including protease, β-1,3-glucanase, and chitinase, was assessed using qualitative plate assays with minor modifications. Protease activity was evaluated on skim milk agar (Kamala and Indira, 2011), β-1,3-glucanase activity on laminarin agar (Koteshwara *et al.*, 2021), and chitinase activity on colloidal chitin agar (Akanksha *et al.*, 2025). Mycelial plugs (5 mm in diameter) were excised from the actively growing margins of 7-day-old colonies, transferred onto the respective medium, and incubated at 25°C for 7 days. To visualize β-1,3-glucanase and chitinase activities, plates were flooded with 0.1% (w/v) Congo red for 15 min, destained with 1 M NaCl for 10 min, and rinsed with SDW. Enzymatic activity was indicated by the formation of clear halos around the colonies, reflecting substrate degradation.

### Microscopy of leaf colonization by GUSF-66

The colonization of cucumber cotyledons by GUSF-66 was observed as described by Nowicki *et al.* (2012) with minor modifications. The cotyledons were treated with the conidial suspension of GUSF-66 and then incubated on water agar in a Petri dish. Three days post-treatment (dpt), cotyledon samples were cleared in a mixture of 100% ethanol and chloroform (3:1, v/v) for 1 day, with the clearing solution being replaced periodically during this period. The cleared samples were then rehydrated in a graded ethanol series (100%, 75%, and 50% ethanol) for 5 min each. The samples were stained with lactophenol trypan blue via boiling in a water bath for 2 min. After cooling at room temperature for 1 h, the samples were transferred to a chloral hydrate solution (2.5 g mL^–1^) for 1 h for further clearing. The stained samples were observed using a BZ-X810 microscope.

### DNA extraction, PCR, sequencing, and phylogenetic anal­ysis

Genomic DNA was extracted from 7-day-old cultures grown on PSA using the NucleoSpin Microbial DNA Extraction Kit (Macherey-Nagel GmbH & Co. Kg) according to the manufacturer’s instructions. Three genetic loci, namely, the internal transcribed spacer (ITS) region, the RNA polymerase II second largest subunit gene (*RPB2*), and the translation elongation factor 1-alpha gene (*TEF1-α*), were amplified by PCR. The ITS region was amplified using the primer pair ITS5/ITS4 (White *et al.*, 1990). The *RPB2* and *TEF-1α* loci were amplified using the primer pairs RPB2-5f2/fRPB2-7cR (Zhao *et al.*, 2023) and EF1-728F/EF1-986R (Carbone and Kohn, 1999), respectively. Thermal cycling conditions consisted of an initial denaturation step at 94°C for 3 min; 35 cycles of denaturation at 94°C for 30 s, annealing at 55°C (ITS and *RPB2*) or 57°C (*TEF1-α*) for 30 s, and extension at 72°C for 1 min; and final extension at 72°C for 5 min, as described by White *et al.* (1990) with minor modifications. PCR products were purified using the NucleoSpin Gel and PCR Clean-up Kit (Macherey-Nagel) according to the manufacturer’s instructions. Sequencing reactions were performed using the BigDye Terminator v3.1 Cycle Sequencing Kit (Thermo Fisher Scientific) with the same primer pairs. Sequencing was conducted following the manufacturer’s protocol with minor modifications, namely, initial denaturation at 96°C for 1 min, followed by 30 cycles of denaturation at 96°C for 10 s, annealing at 50°C for 5 s, and extension at 60°C for 2.5 min. The resulting sequences were assembled and edited using standard procedures, and the sequences obtained were deposited in the DDBJ database.

Regarding species identification, the sequences obtained were compared via BLAST searches using reference sequences available in GenBank. Phylogenetic anal­yses were performed using a concatenated dataset of ITS, *RPB2*, and *TEF1-α* sequences in MEGA version 12.0. A maximum likelihood tree was constructed using the Kimura two-parameter model, and branch support was evaluated by a bootstrap anal­ysis with 1,000 replicates (Kumar *et al.*, 2024).

### Potential pathogenicity of GUSF-66 against soybean plants

Soybean seeds (*Glycine max* (L.) Merr. cv Enrei) were surface-sterilized via immersion in 70% (v/v) ethanol for 1 min, followed by 1% (w/v) sodium hypochlorite for 1 min, and were subsequently rinsed three times with SDW. The sterilized seeds were germinated on moist filter paper at 28°C for 2 days and immersed in the conidial suspension of GUSF-66 (approximately 1×10^7^ conidia mL^–1^) for 30 min. Seeds immersed in 0.01% (v/v) Silwet L-77 solution served as the control. The seeds were then sown in 9-cm plastic pots containing a double-autoclaved soil mixture of commercial soil and autoclaved vermiculite at a 1:1 (v/v) ratio. Pots were placed in a controlled-environment chamber under a 12-‍h/12-h light/dark cycle at 27°C for the first 3 days, after which the temperature was adjusted to 23°C for continued growth.

Thirty days after sowing, the soybean plants were uprooted and exami­ned for symptoms, such as discoloration, blackening, and soft rot of the taproots and lateral roots, as well as the yellowing of leaves (Zhao *et al.*, 2024). Additionally, the dry weight of plants was measured.

### Statistical anal­ysis

All statistical anal­yses were performed using the XLSTAT software program (Addinsoft). Percentage data were arcsine square root-transformed before the statistical anal­ysis. Differences between the treatment and control groups were analyzed using a two-tailed, independent-samples *t*-test. Conversely, differences among three or more treatments were analyzed by a one-way anal­ysis of variance followed by Tukey’s honestly significant difference test. The significance of differences was indicated by *P*<0.05. Figures present non-transformed data.

## Results

### Isolation of epiphytic and endophytic fungi

In total, 137 fungi were isolated from plant leaves, 108 of which produced spores on PSA medium. Of these spore-forming strains, 89 were considered to be epiphytes because they were isolated from the leaf segment surface, and the remaining 19 strains were considered to be endophytes because they were recovered from surface-sterilized leaf segments (Table S1). The spore-producing strains were designated as the GUSF series and subjected to a subsequent detached cotyledon assay.

### Detached cucumber cotyledon assay

As preliminary screening, we evaluated the biocontrol activities of the GUSF strains against anthracnose using a detached cucumber cotyledon assay. Large necrotic lesions were produced on the control cotyledons when challenged with *C. orbiculare* (Fig. 1). Conversely, lesion development was suppressed to varying degrees by more than half of the‍ ‍GUSF strains, although some strains enhanced lesion development (Fig. S1). Of the 108 tested strains capable of sporulation, GUSF-44, GUSF-66, and GUSF-153 most effectively suppressed lesion development through repeated experiments, with an average reduction exceeding 60% (Fig. 1, Table 1). These strains were selected as primary candidates.

### Pot experiments

The efficacy of the primary candidate strains against anthracnose was further tested on the true leaves of pot-grown cucumber plants. Symptoms became visible at nearly all the inoculated sites on the control leaves at 4–5 dpi, and large necrotic lesions had developed by 7 dpi (Fig. 2). Although symptoms appeared on leaves treated with the candidate strains at 4–5 dpi, the number of symptoms was lower than that of control plants (Table 2). Necrotic lesions at 7 dpi were smaller in size in treated plants (Fig. 2). Among the GUSF treatments, GUSF-66 achieved the greatest reduction in the lesion area versus the control (73%; Table 2). Based on these results, GUSF-66 was selected as the most promising candidate.

### Morphological characteristics of GUSF-66

After an incubation on PSA in the dark, GUSF-66 formed yellowish-white to pale-yellow colonies with localized green areas corresponding to conidial masses. The colony surface was cottony to felty with abundant aerial mycelia (Fig. 3A). Conidiophores were produced throughout the colonies. Primary conidiophores were verticillium-like, bearing divergent phialides that were straight, cylindrical, and slightly tapered toward the apex (Fig. 3B). Secondary conidiophores were also observed, possessing one or two branches that diverged and terminated in moderately divergent metulae bearing appressed phialides. The phialides were nearly cylindrical, tapering toward the upper region, and they were straight to slightly curved (Fig. 3C). Conidia were hyaline, ellipsoidal, and slightly curved, with one side appearing almost straight (Fig. 3D).

### *In vitro* antagonism

As presented in Fig. 4, GUSF-66 inhibited the mycelial growth of *C. orbiculare* on PSA medium by 54.23±2.41%. Furthermore, GUSF-66 mycelia actively overgrew the pathogen colony.

### *In vitro* interaction between *C. orbiculare* and GUSF-66

In the control treatment, *C. orbiculare* conidia actively germinated on water agar (Fig. 5A), whereas *C. orbiculare* conidial germination was inhibited when co-cultured with GUSF-66 (Fig. 5B). The conidial germination rate reached approximately 87% by 1 dpi in the control treatment. Although GUSF-66 did not strongly inhibit germination, the average rate was lower than that of the control group (approximately 76%; Fig. 5C). As shown in Fig. 5B, conidial germination was suppressed in the absence of direct physical contact between GUSF-66 hyphae and *C. orbiculare* conidia.

### Enzymatic activity of GUSF-66

As demonstrated in Fig. 6, GUSF-66 produced distinct, clear zones around the colonies grown on skim milk agar, laminarin agar, and colloidal chitin agar, demonstrating the production of protease, β-1,3-glucanase, and chitinase, respectively.

### Leaf colonization by GUSF-66

No visible pathological changes, including necrosis and chlorosis, were observed on the cotyledon treated with GUSF-66 at 3 dpt (Fig. 7A). Additionally, microscopy of the adaxial surface of GUSF-66-treated cotyledons before clearing revealed no apparent pathological changes, such as browning or collapse, in epidermal cells (Fig. 7B). After the samples had been stained with lactophenol trypan blue, most of the GUSF-66 conidia had germinated and were producing actively elongating hyphae on the cotyledon surface (Fig. 7C). No visible damage or defense reactions were observed in epidermal cells beneath the GUSF-66 hyphae. Overall, these results suggest that the strain established epiphytic colonization within 3 days after its application without exerting harmful effects on cucumber plants.

### Identification of GUSF-66

Preliminary sequence similarity searches indicated that GUSF-66 belonged to the genus *Clonostachys*. To identify its taxonomic position, a multilocus phylogenetic anal­ysis was conducted using concatenated ITS, *RPB2*, and *TEF1-α* sequences. In the maximum-likelihood tree, GUSF-66 clustered within the *C. chloroleuca* clade alongside the reference strains CBS:141588, CCLF677, and CCLF678 with strong bootstrap support (90%). This clade was resolved separately from the closely related *C. rhizophaga* clade, which had 91% bootstrap support (Fig. 8). These results identified GUSF-66 as *C. chloroleuca*. The ITS, *RPB2*, and *TEF1-α* sequences of GUSF-66 were deposited in GenBank under the accession numbers LC936069, LC936070, and LC936071, respectively.

### The potential pathogenicity of GUSF-66 against soybean plants

Soybean plants treated with GUSF-66 exhibited no visible symptoms, such as leaf yellowing in their shoots, and their growth was similar to that of the control plants (Fig. S2A). Similarly, the root systems of the GUSF-66–treated plants were similar to those of the control plants. No discoloration, blackening, or soft rot was observed in the taproots or lateral roots of GUSF-66–treated plants (Fig. S2A). Additionally, the dry weights of shoots and roots did not significantly differ between the treatments (Fig. S2B), suggesting that GUSF-66 is non-pathogenic to soybean plants.

## Discussion

The present study aimed to identify plant-associated fungi with biocontrol activity against cucumber anthracnose. GUSF-66 exhibited the most consistent and pronounced disease-suppressive activity throughout the screening experiments, and it was selected as a promising BCA candidate against cucumber anthracnose. Although GUSF-66 was originally isolated as an epiphyte from *H. brasiliensis*, the strain was also capable of colonizing cucumber cotyledon surfaces within 3 days after its application. Since *C. orbiculare* initiates infection on aerial cucumber tissues, epiphytic fungi adapted to the phyllosphere may represent particularly suitable BCAs for suppressing anthracnose development on leaf surfaces.

The multilocus phylogenetic anal­ysis based on the ITS, *RPB2*, and *TEF1-α* regions identified GUSF-66 as *C. chloroleuca*. This was further supported by observations of macromorphological and microscopic characteristics. The cultural characteristics and structures of the primary and secondary conidiophores and conidia of GUSF-66 matched the descriptions of *C. chloroleuca* first reported by Moreira *et al.* (2016). *Clonostachys* species are ubiquitous fungi with diverse ecological lifestyles, including saprotrophic, endophytic, epiphytic, and mycoparasitic associations (Piombo *et al.*, 2023; Zhao *et al.*, 2023). Members of this genus have been widely recognized as potential BCAs against fungal plant pathogens (Sun *et al.*, 2020), plant-parasitic nematodes (Shravani *et al.*, 2026), and insect herbivores (Iqbal *et al.*, 2018). Previous studies on the biocontrol activity of *Clonostachys* species mainly focused on soilborne pathogens and rhizosphere-associated applications, particularly those involving *C. rosea* (formerly classified as *Gliocladium roseum*) (Chatterton *et al.*, 2008; Sun *et al.*, 2023; Venkatesan *et al.*, 2023; Stucky *et al.*, 2024). Nevertheless, the effectiveness of *Clonostachys* species, including *C. chloroleuca*, obtained from soil and plants against foliar diseases, such as gray mold caused by *Botrytis cinerea* and Fusarium head blight caused by *Fusarium graminearum* has been demonstrated (Hasan *et al.*, 2022; Tai *et al.*, 2026). Conversely, although some studies reported the *in vitro* antagonistic activity of *Clonostachys* species against *Colletotrichum* species (Belingheri Lagunes *et al.*, 2024), their effectiveness against anthracnose disease *in planta* remains unclear. Therefore, to the best of our knowledge, this study provides the first evidence to show that an epiphytic *C. chloroleuca* strain may effectively suppress cucumber anthracnose caused by *C. orbiculare*.

Mycoparasitism is widely recognized as an important mechanism underlying the biocontrol activity of *Clonostachys* species against fungal pathogens (Hasan *et al.*, 2022; Yu *et al.*, 2023). *C. chloroleuca* has also been reported to parasitize plant pathogenic fungi, such as *Sclerotinia sclerotiorum*, *B. cinerea*, *Alternaria grandis*, and *Rhizoctonia solani* (da Silva *et al.*, 2021; Hasan *et al.*, 2022; Lv *et al.*, 2023; Sun *et al.*, 2023). However, although GUSF-66 produced hydrolytic enzymes, including chitinase, β-1,3-glucanase, and protease, which are associated with fungal cell wall degradation during mycoparasitism (Chatterton and Punja, 2009; Lv *et al.*, 2023), it did not exhibit clear mycoparasitic interactions with *C. orbiculare* during the co-culture on water agar. Conversely, GUSF-66 reduced *C. orbiculare* conidial germination without direct contact on water agar and inhibited *C. orbiculare* hyphal growth on PSA. These results suggest the involvement of diffusible antifungal factors in the suppressive activity of GUSF-66. The similar inhibition of early infection processes has been reported for *C. rosea* IK726, which suppressed conidial germination and appressorium formation by *Bipolaris sorokiniana* on barley leaves (Jensen *et al.*, 2016). *Clonostachys* species have been shown to produce various antifungal metabolites, including arthrichitin and cyclosporin A (Han *et al.*, 2020). Therefore, extracellular lytic enzymes and diffusible antifungal metabolites may both contribute to *C. orbiculare* suppression by GUSF-66. Although the present study did not directly observe *in planta* interactions between GUSF-66 and *C. orbiculare*, GUSF-66 may interfere with the early infection processes of *C. orbiculare* on leaf surfaces by secreting antifungal factors.

Furthermore, host-mediated effects may contribute to the protective activity of GUSF-66. In the pot experiment, the GUSF-66 treatment did not significantly reduce the number of lesions, but suppressed lesion expansion. This pattern suggests that GUSF-66 restricted pathogen development after infection establishment. Induced resistance conferred by BCAs often manifests as the suppression of lesion expansion rather than the complete inhibition of infection establishment (Van Loon, 1976; Van Loon *et al.*, 1998). Therefore, one possible explanation is the involvement of host defense responses associated with the GUSF-66 treatment. Previous studies demonstrated that some *Clonostachys* strains stimulate host immunity, thereby suppressing fungal diseases (Gong *et al.*, 2017). However, further molecular and histological anal­yses are necessary to‍ ‍clarify the contribution of host-mediated mechanisms to disease suppression.

Although *Clonostachys* species are generally regarded as beneficial fungi, several species have been reported to cause localized damage or root rot in specific crops (Bienapfl *et al.*, 2012; Diaz *et al.*, 2022; Gautheron *et al.*, 2025). *C. chloroleuca* has also been implicated in the root rot of soybean (Zhao *et al.*, 2024), cassava (Madrassi *et al.*, 2023), and *Ophiopogon japonicus* (Wang *et al.*, 2026), as well as in chickpea wilt (Cota-Barreras *et al.*, 2022). These studies highlight the importance of a strain-level safety evaluation when developing *C. chloroleuca* as a BCA. Therefore, we evaluated the pathogenicity of GUSF-66 toward soybean, an important crop in Japan, and showed that the strain caused no visible disease symptoms under the conditions tested. Although further safety assessments using additional plant species and environmental conditions are necessary, this result provides preliminary support for the development of GUSF-66 as a biofungicide.

Overall, the present study highlights *C. chloroleuca* GUSF-66 as a promising BCA against cucumber anthracnose caused by *C. orbiculare*. The strain exhibited multiple antagonistic characteristics, including the suppression of conidial germination and hyphal growth, the production of extracellular lytic enzymes, and the successful colonization of cucumber cotyledon surfaces. To the best of our knowledge, this is the first study to report the biocontrol potential of *C. chloroleuca* against cucumber anthracnose. These results suggest that phyllosphere-associated fungi represent valuable sources of BCAs against foliar pathogens. Further studies are needed to clarify the *in planta* interactions between GUSF-66 and *C. orbiculare*, identify the antifungal factors involved in disease suppression, and evaluate the efficacy and stability of GUSF-66 under greenhouse and field conditions.

## Citation

Kusdiana, A. P. J., Hayakawa, Y., Tumewu, S. A., Suga, H., and Shimizu, M. (2026) A Novel Epiphytic *Clonostachys chloroleuca* Strain that Effectively Controls Cucumber Anthracnose. *Microbes Environ ***41**: ME26058.

https://doi.org/10.1264/jsme2.ME26058

## Supplementary Material

Supplementary Material

## Figures and Tables

**Fig. 1.**
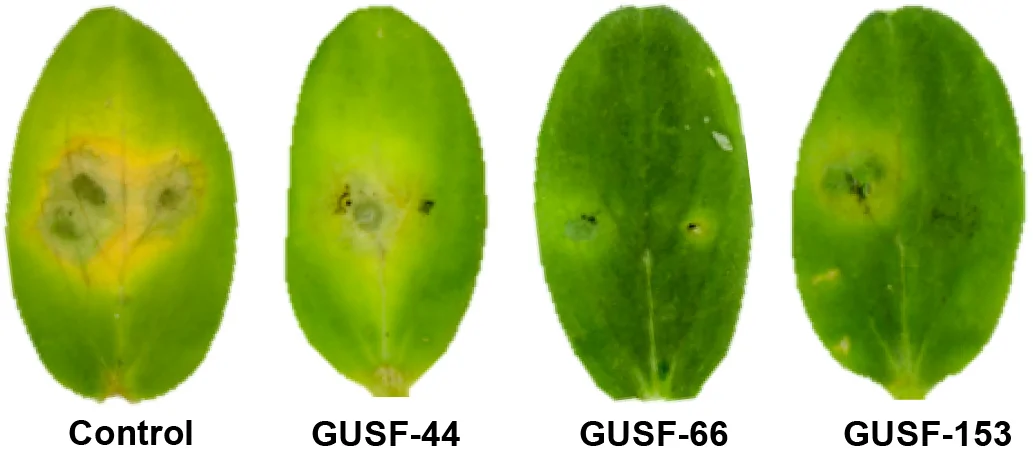
Suppression of anthracnose symptom development by a pretreatment with primary candidate strains in the detached cotyledon assay. Cucumber cotyledons were pretreated with conidial suspensions of the primary candidate strains before the pathogen inoculation. Control cotyledons were treated with 0.01% Silwet L-77 (v/v). Photographs were taken at 7 dpi.

**Fig. 2.**
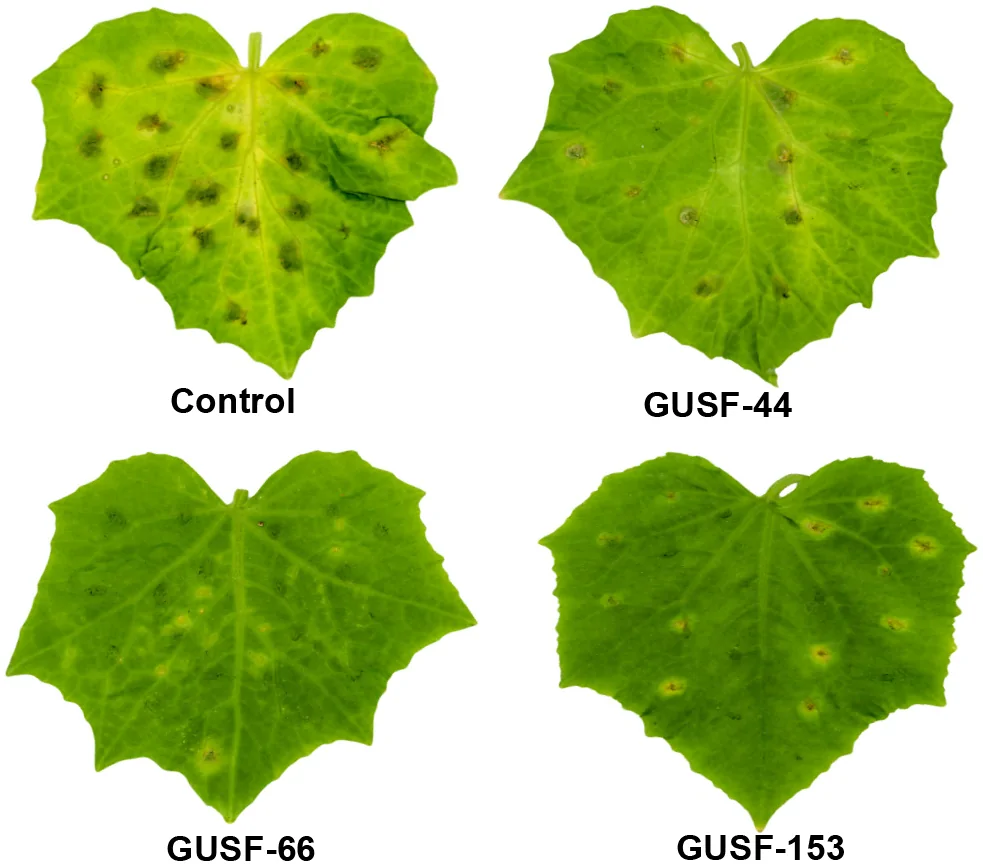
Suppressive effects of primary candidate strains against anthracnose on true leaves of pot-grown cucumber plants. The second true leaf of each plant was sprayed with conidial suspensions of the selected GUSF strains 1 day before the pathogen inoculation. Control leaves were sprayed with 0.01% Silwet L-77. Photographs were taken at 7 dpi.

**Fig. 3.**
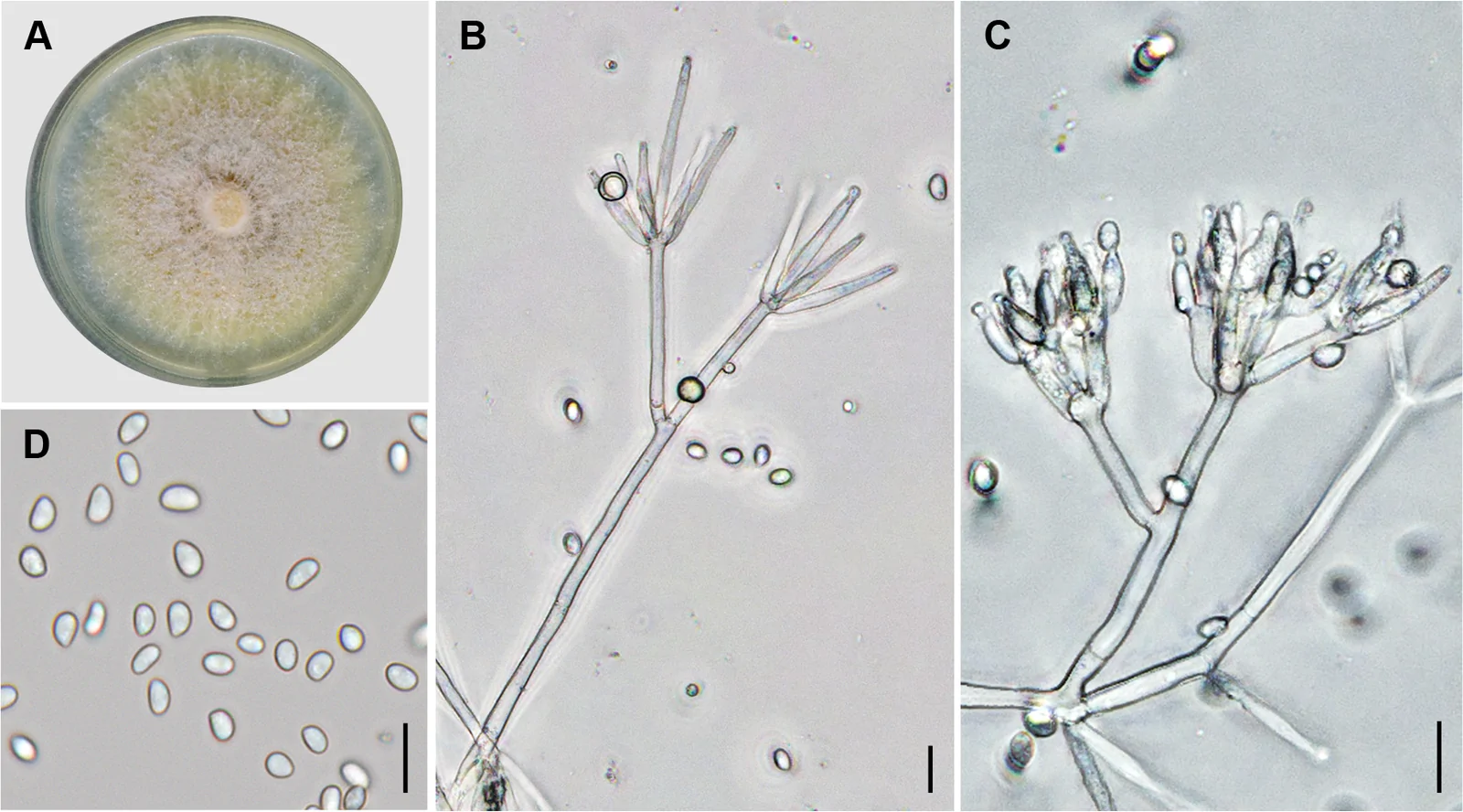
Morphological characteristics of GUSF-66. (A) Thirty-day-old colonies incubated at 25°C on PSA. (B) Primary conidiophore with divergent branches and phialides. (C) Secondary conidiophores with divergent primary branches, terminating in adpressed phialides. (D) Conidia. Scale bar=10 μm.

**Fig. 4.**
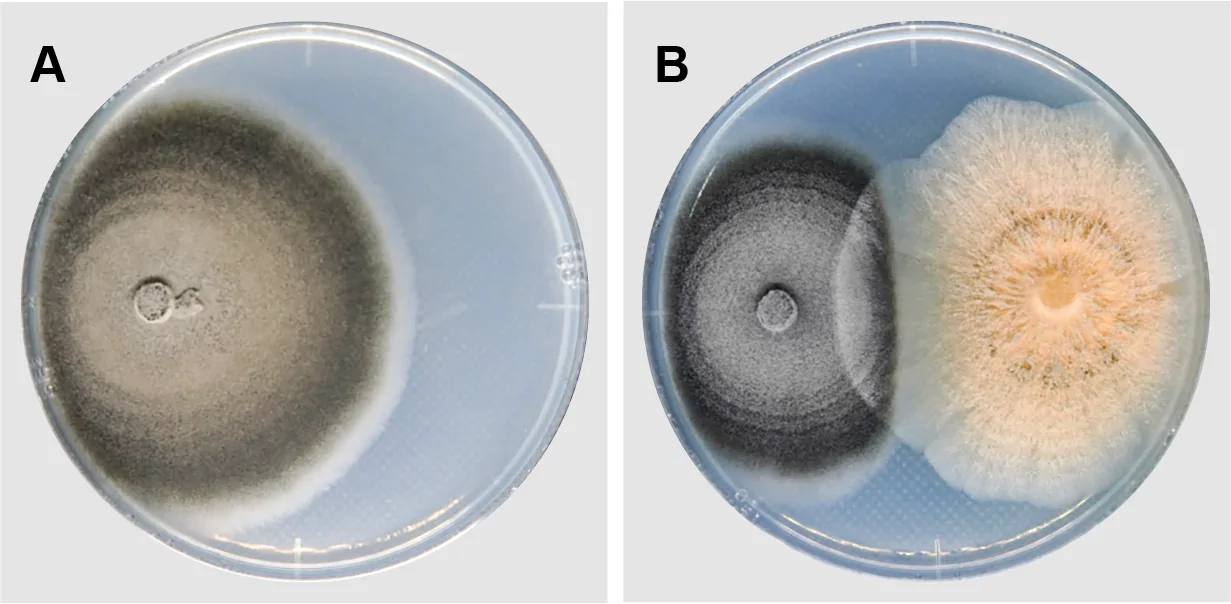
Antagonistic activity of GUSF-66 against *Colletotrichum orbiculare* 104-T in a dual culture assay. Photographs were taken at 10 dpi. (A) *C. orbiculare* 104-T cultured alone. (B) *C. orbiculare* 104-T (left) co-cultured with GUSF-66 (right).

**Fig. 5.**
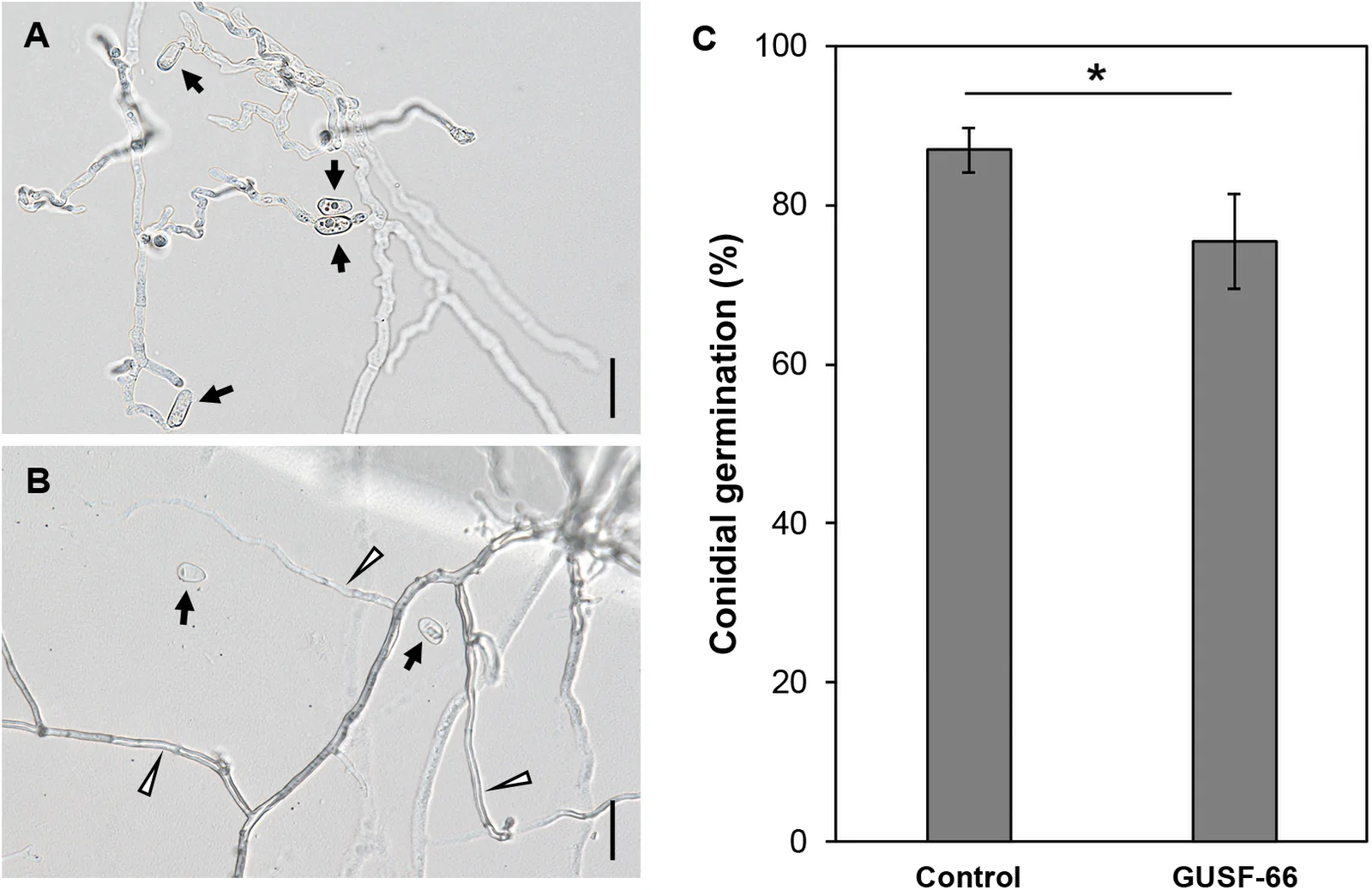
Effects of GUSF-66 on the germination of *Colletotrichum orbiculare* 104-T conidia. (A, B) *C. orbiculare* conidia in the (A) control and (B) GUSF-66 treatments, respectively. Photographs were taken at 2 dpi. Black arrows indicate *C. orbiculare* conidia, and white arrowheads indicate GUSF-66 hyphae. Scale bars=20 μm. (C) Conidial germination rate of *C. orbiculare*. Data are presented as the mean±SD. Statistical anal­yses were performed using arcsine square root-transformed data; however, non-transformed percentage values are presented. An asterisk indicates a significant difference at *P*<0.05 based on a two-tailed independent-samples *t*-test.

**Fig. 6.**
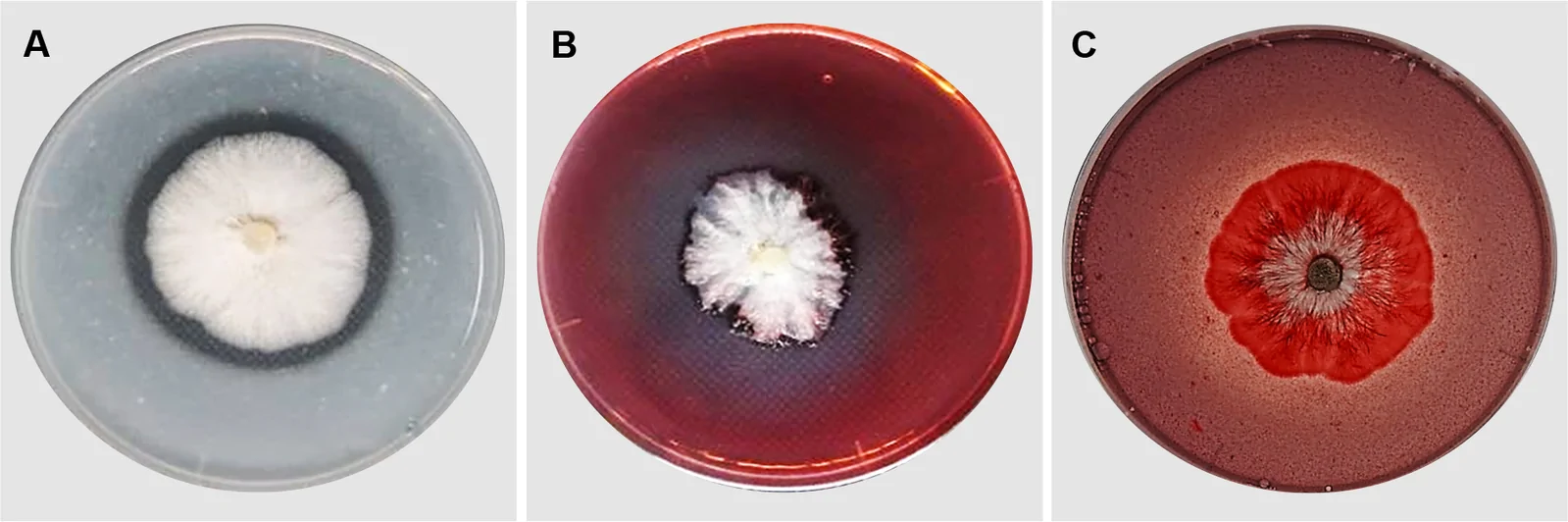
Extracellular hydrolytic enzyme activity of GUSF-66 on substrate-specific media. Clear zones formed around GUSF-66 colonies on (A) skim milk agar, (B) laminarin agar, and (C) colloidal chitin agar, corresponding to protease, β-1,3-glucanase, and chitinase activities, respectively. Images were captured after 7 days of incubation.

**Fig. 7.**
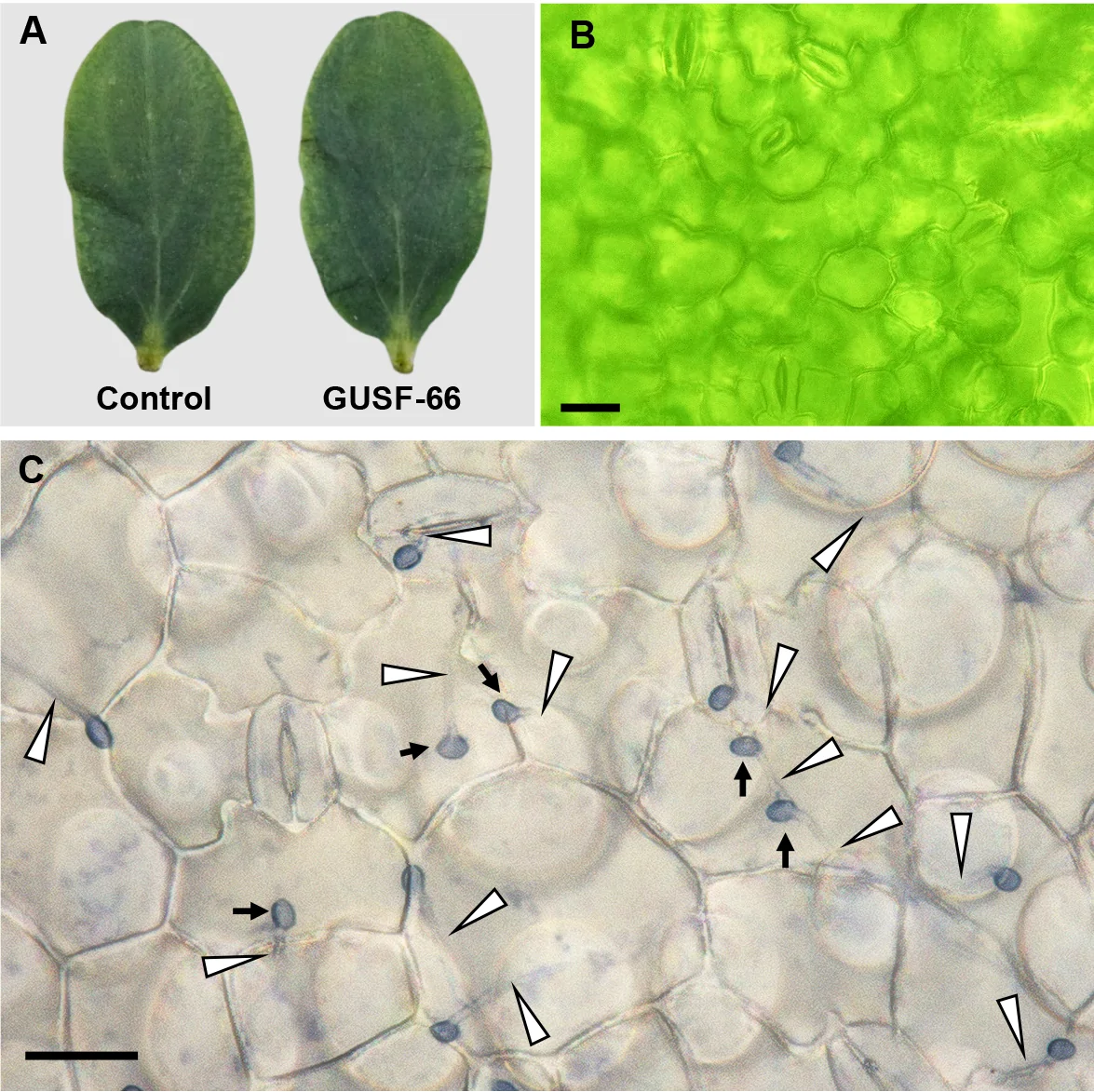
Asymptomatic epiphytic colonization of cucumber cotyledons by GUSF-66. (A) Images of the adaxial side of cotyledons in the control treatment (left) and GUSF-66 treatment (right). (B) A micrograph of the surface of a GUSF-66-treated cotyledon before clearing. (C) A micrograph of a cotyledon stained with lactophenol trypan blue, showing GUSF-66 colonization of the cotyledon surface. Black arrows indicate the conidia, and white arrowheads indicate the germ tubes/hyphae of GUSF-66. The photo was taken 3 days post-treatment. Scale bar=20 μm.

**Fig. 8.**
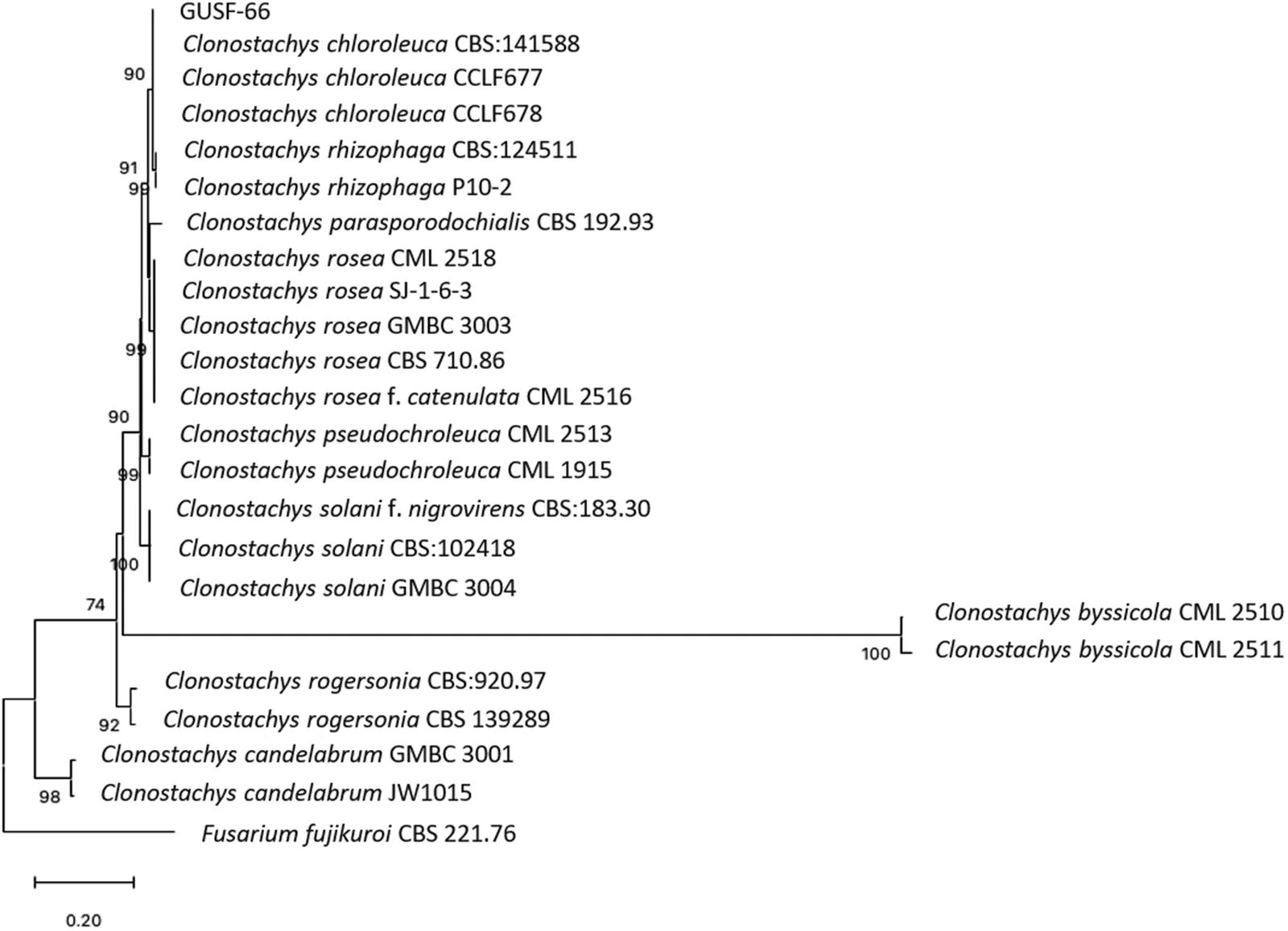
Maximum likelihood phylogenetic tree of *Clonostachys* spp. based on concatenated ITS, *RPB2*, and *TEF1-α* sequences. GUSF-66 clustered within the *Clonostachys chloroleuca* clade with 90% bootstrap support and was separated from the *C. rhizophaga* clade with 91% bootstrap support. Bootstrap values are shown at the nodes. *Fusarium fujikuroi* was used as the outgroup.

**Table 1.** Primary candidate fungal strains selected according to their ability to reduce the *Colletotrichum orbiculare* lesion area in detached cucumber cotyledon assays

Strain	Lesion area reduction (%)
GUSF-44	62.64±7.85
GUSF-66	61.21±13.15
GUSF-153	71.12±11.20

**Table 2.** Primary candidate fungal strains selected according to their ability to reduce the *Colletotrichum orbiculare* lesion area in potted cucumber plants

Strain	No. of lesions/leaf	Total lesion area/leaf (cm^2^)
Control	11.82±5.05 a	2.19±2.09 a
GUSF-44	8.67±5.97 a	1.21±1.07 ab
GUSF-66	7.57±6.29 a	0.59±0.62 b
GUSF-153	10.07±5.75 a	1.10±1.00 ab

Values are presented as means±SD. Statistical comparisons were performed separately for each column. Different lowercase letters within each column indicate significant differences among treatments, as assessed by Tukey’s honestly significant difference test following a one-way ANOVA (*P*<0.05).
