# Supplementary material for: A Novel Epiphytic Clonostachys chloroleuca Strain that Effectively Controls Cucumber Anthracnose
Source: Microbes Environ. 2026 Sep 29;41(3):ME26058. doi: 10.1264/jsme2.ME26058 (PMC13659348; doi:10.1264/jsme2.ME26058)
Supplement: Supplementary file 1 [file 41_26058_s1.pdf]

Supplementary Table S1. Epiphytic and endophytic fungi isolated from the leaves of seven different plant species

Isolate code*	Host plant	Origin	Sporulation
GUSF-4	*Hevea brasiliensis*	Epiphytic	Present
GUSF-5	*Hevea brasiliensis*	Epiphytic	Present
GUSF-6	*Hevea brasiliensis*	Epiphytic	Present
GUSF-9	*Hevea brasiliensis*	Epiphytic	Present
GUSF-11	*Hevea brasiliensis*	Epiphytic	Present
GUSF-14	*Hevea brasiliensis*	Epiphytic	Present
GUSF-15	*Hevea brasiliensis*	Epiphytic	Present
GUSF-18	*Hevea brasiliensis*	Epiphytic	Present
GUSF-19	*Hevea brasiliensis*	Epiphytic	Present
GUSF-20	*Hevea brasiliensis*	Epiphytic	Absent
GUSF-21	*Hevea brasiliensis*	Epiphytic	Absent
GUSF-22	*Hevea brasiliensis*	Epiphytic	Absent
GUSF-23	*Hevea brasiliensis*	Epiphytic	Absent
GUSF-24	*Hevea brasiliensis*	Epiphytic	Absent
GUSF-25	*Hevea brasiliensis*	Epiphytic	Absent
GUSF-26	*Hevea brasiliensis*	Epiphytic	Absent
GUSF-27	*Hevea brasiliensis*	Epiphytic	Absent
GUSF-28	*Hevea brasiliensis*	Epiphytic	Absent
GUSF-29	*Hevea brasiliensis*	Epiphytic	Absent
GUSF-30	*Hevea brasiliensis*	Epiphytic	Present
GUSF-32	*Hevea brasiliensis*	Epiphytic	Present
GUSF-34	*Hevea brasiliensis*	Epiphytic	Present
GUSF-37	*Hevea brasiliensis*	Epiphytic	Present
GUSF-38	*Hevea brasiliensis*	Epiphytic	Present
GUSF-41	*Hevea brasiliensis*	Epiphytic	Present
GUSF-42	*Hevea brasiliensis*	Epiphytic	Present
GUSF-43	*Hevea brasiliensis*	Epiphytic	Present

GUSF-44	*Hevea brasiliensis*	Epiphytic	Present
GUSF-47	*Hevea brasiliensis*	Epiphytic	Present
GUSF-48	*Hevea brasiliensis*	Epiphytic	Present
GUSF-49	*Hevea brasiliensis*	Epiphytic	Present
GUSF-50	*Hevea brasiliensis*	Epiphytic	Present
GUSF-51	*Hevea brasiliensis*	Epiphytic	Present
GUSF-52	*Hevea brasiliensis*	Epiphytic	Present
GUSF-54	*Hevea brasiliensis*	Epiphytic	Absent
GUSF-55	*Hevea brasiliensis*	Epiphytic	Absent
GUSF-56	*Hevea brasiliensis*	Epiphytic	Absent
GUSF-57	*Hevea brasiliensis*	Epiphytic	Absent
GUSF-58	*Hevea brasiliensis*	Epiphytic	Absent
GUSF-59	*Hevea brasiliensis*	Epiphytic	Absent
GUSF-60	*Hevea brasiliensis*	Epiphytic	Absent
GUSF-61	*Hevea brasiliensis*	Epiphytic	Absent
GUSF-62	*Hevea brasiliensis*	Endophytic	Present
GUSF-63	*Hevea brasiliensis*	Endophytic	Present
GUSF-64	*Hevea brasiliensis*	Endophytic	Present
GUSF-65	*Hevea brasiliensis*	Endophytic	Present
GUSF-66	*Hevea brasiliensis*	Epiphytic	Present
GUSF-67	*Hevea brasiliensis*	Epiphytic	Present
GUSF-68	*Hevea brasiliensis*	Epiphytic	Present
GUSF-69	*Hevea brasiliensis*	Epiphytic	Present
GUSF-70	*Hevea brasiliensis*	Epiphytic	Present
GUSF-71	*Hevea brasiliensis*	Epiphytic	Present
GUSF-72	*Hevea brasiliensis*	Epiphytic	Present
GUSF-74	*Hevea brasiliensis*	Epiphytic	Present
GUSF-75	*Hevea brasiliensis*	Epiphytic	Present
GUSF-77	*Hevea brasiliensis*	Epiphytic	Present
GUSF-78	*Hevea brasiliensis*	Epiphytic	Present
GUSF-79	*Hevea brasiliensis*	Epiphytic	Present

GUSF-80	*Hevea brasiliensis*	Epiphytic	Present
GUSF-82	*Hevea brasiliensis*	Epiphytic	Present
GUSF-83	*Hevea brasiliensis*	Epiphytic	Absent
GUSF-84	*Hevea brasiliensis*	Epiphytic	Absent
GUSF-85	*Hevea brasiliensis*	Epiphytic	Absent
GUSF-86	*Hevea brasiliensis*	Epiphytic	Absent
GUSF-87	*Hevea brasiliensis*	Epiphytic	Absent
GUSF-88	*Hevea brasiliensis*	Epiphytic	Absent
GUSF-89	*Hevea brasiliensis*	Epiphytic	Absent
GUSF-91	*Hevea brasiliensis*	Epiphytic	Present
GUSF-92	*Hevea brasiliensis*	Epiphytic	Present
GUSF-93	*Hevea brasiliensis*	Epiphytic	Present
GUSF-95	*Hevea brasiliensis*	Epiphytic	Present
GUSF-96	*Hevea brasiliensis*	Epiphytic	Absent
GUSF-97	*Mallotus* sp.	Endophytic	Present
GUSF-98	*Mallotus* sp.	Endophytic	Present
GUSF-101	*Mallotus* sp.	Endophytic	Present
GUSF-104	*Mallotus* sp.	Endophytic	Present
GUSF-115	*Mallotus* sp.	Endophytic	Present
GUSF-117	*Mallotus* sp.	Epiphytic	Absent
GUSF-118	*Euphorbia trigona*	Epiphytic	Present
GUSF-120	*Euphorbia trigona*	Epiphytic	Present
GUSF-121	*Euphorbia trigona*	Epiphytic	Present
GUSF-122	*Codiaeum variegatum*	Endophytic	Present
GUSF-123	*Codiaeum variegatum*	Endophytic	Present
GUSF-124	*Codiaeum variegatum*	Endophytic	Present
GUSF-125	*Codiaeum variegatum*	Endophytic	Present
GUSF-129	*Codiaeum variegatum*	Epiphytic	Present
GUSF-130	*Codiaeum variegatum*	Epiphytic	Present
GUSF-133	*Mallotus* sp.	Epiphytic	Present
GUSF-135	*Mallotus* sp.	Epiphytic	Present

GUSF-137	*Mallotus* sp.	Epiphytic	Present
GUSF-138	*Mallotus* sp.	Epiphytic	Present
GUSF-141	*Mallotus* sp.	Epiphytic	Present
GUSF-142	*Mallotus* sp.	Epiphytic	Present
GUSF-143	*Manihot esculenta*	Epiphytic	Present
GUSF-144	*Manihot esculenta*	Epiphytic	Present
GUSF-145	*Manihot esculenta*	Epiphytic	Present
GUSF-147	*Manihot esculenta*	Epiphytic	Present
GUSF-148	*Manihot esculenta*	Epiphytic	Present
GUSF-149	*Manihot esculenta*	Epiphytic	Present
GUSF-150	*Manihot esculenta*	Epiphytic	Present
GUSF-151	*Manihot esculenta*	Epiphytic	Present
GUSF-152	*Manihot esculenta*	Epiphytic	Present
GUSF-153	*Manihot esculenta*	Epiphytic	Present
GUSF-154	*Manihot esculenta*	Epiphytic	Present
GUSF-155	*Manihot esculenta*	Epiphytic	Present
GUSF-156	*Manihot esculenta*	Epiphytic	Present
GUSF-157	*Manihot esculenta*	Epiphytic	Present
GUSF-158	*Manihot esculenta*	Endophytic	Present
GUSF-159	*Manihot esculenta*	Endophytic	Present
GUSF-160	*Manihot esculenta*	Endophytic	Present
GUSF-161	*Euphorbia trigona*	Epiphytic	Present
GUSF-162	*Euphorbia trigona*	Epiphytic	Present
GUSF-163	*Euphorbia trigona*	Epiphytic	Present
GUSF-164	*Euphorbia trigona*	Epiphytic	Present
GUSF-165	*Euphorbia trigona*	Epiphytic	Present
GUSF-166	*Ricinus communis*	Epiphytic	Present
GUSF-168	*Ricinus communis*	Epiphytic	Present
GUSF-170	*Ricinus communis*	Epiphytic	Present
GUSF-172	*Ricinus communis*	Epiphytic	Present
GUSF-173	*Ricinus communis*	Epiphytic	Present

GUSF-174	*Ricinus communis*	Epiphytic	Present
GUSF-175	*Ricinus communis*	Epiphytic	Present
GUSF-176	*Ricinus communis*	Epiphytic	Present
GUSF-178	*Ricinus communis*	Epiphytic	Present
GUSF-179	*Ricinus communis*	Epiphytic	Present
GUSF-180	*Ricinus communis*	Epiphytic	Present
GUSF-181	*Ricinus communis*	Endophytic	Present
GUSF-183	*Ricinus communis*	Endophytic	Present
GUSF-184	*Ricinus communis*	Endophytic	Absent
GUSF-185	*Euphorbia pulcherrima*	Epiphytic	Present
GUSF-189	*Euphorbia pulcherrima*	Epiphytic	Present
GUSF-190	*Euphorbia pulcherrima*	Epiphytic	Present
GUSF-191	*Euphorbia pulcherrima*	Epiphytic	Present
GUSF-194	*Euphorbia pulcherrima*	Epiphytic	Present
GUSF-195	*Euphorbia pulcherrima*	Epiphytic	Present
GUSF-196	*Euphorbia pulcherrima*	Epiphytic	Absent
GUSF-197	*Euphorbia pulcherrima*	Endophytic	Present

Fig. S1.

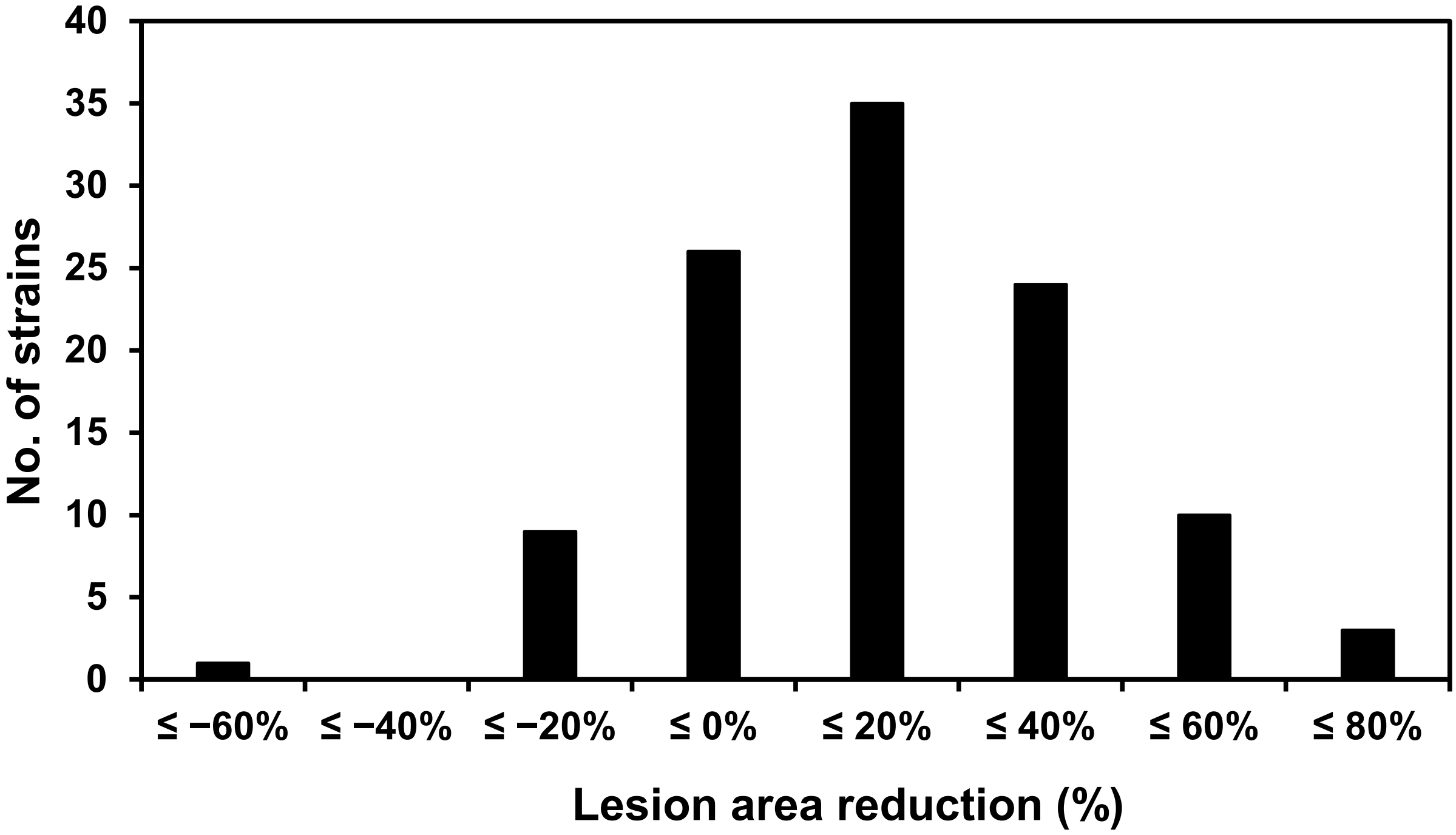

Fig. S1. Histogram presenting the distribution of lesion area reduction (LAR) induced by GUSF strains evaluated for biocontrol activity against cucumber anthracnose using a detached cotyledon assay. The x-axis presents LAR (%) categories, and the y-axis indicates the number of strains within each category.

Fig. S2.

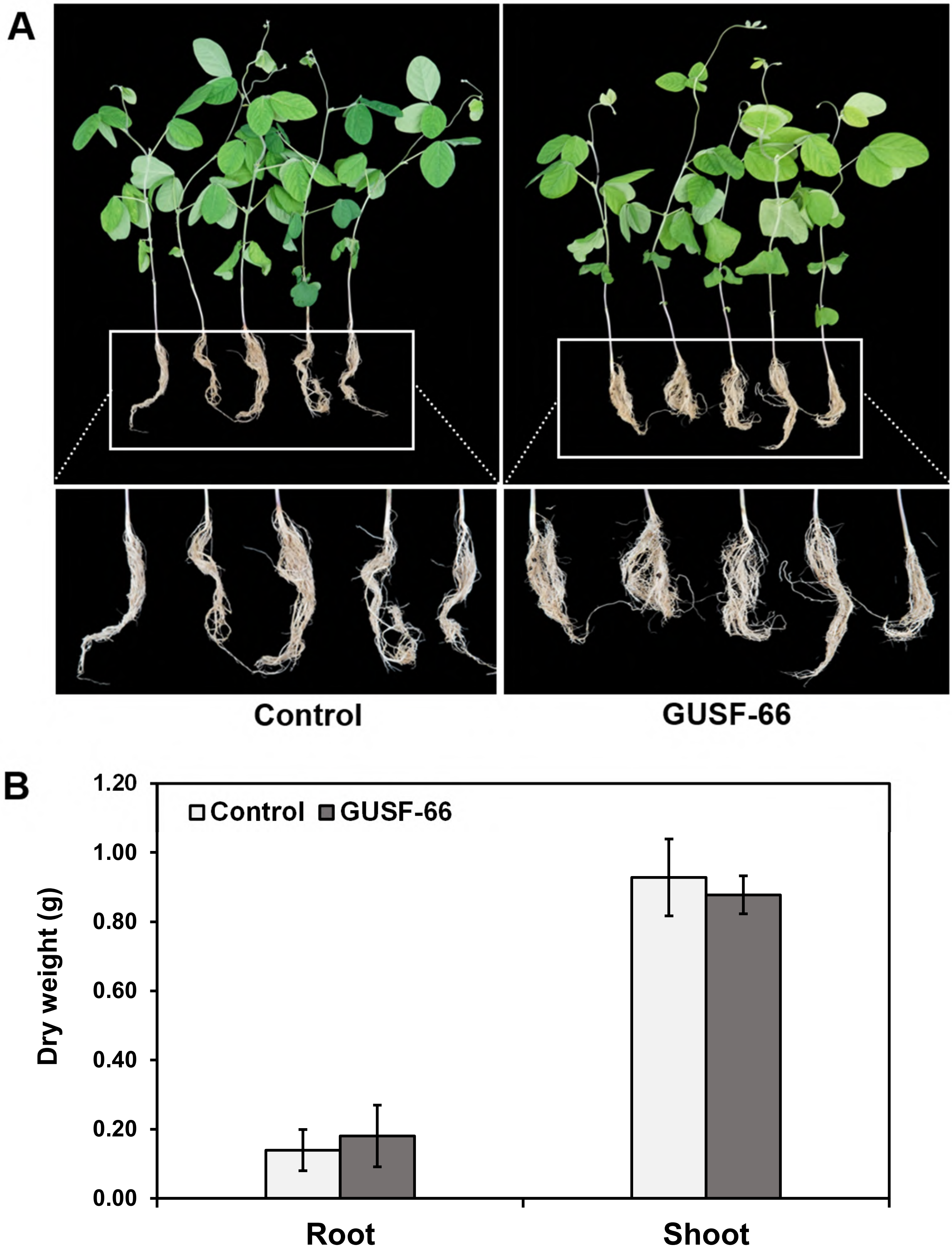

Figure S2. Pathogenicity of *Clonostachys chloroleuca* GUSF-66 against soybean seedlings. (A) Whole-plant morphology and root systems of soybean seedlings treated with GUSF-66 and the control. No visible disease symptoms or growth abnormalities were observed in GUSF-66–treated plants at 30 days after inoculation. (B) Dry weights of roots and shoots at 30 days after inoculation. No significant differences were detected between GUSF-66–treated and control plants according to a two-tailed independent-samples *t*-test ($p > 0.05$).
